# Personal

**Published:** 1911-08

**Authors:** 


### PERSONAL

(The editors would be glad to receive items for this department from any physician, whether a subscriber or not. In the future, we shall rely largely upon notices of removal, change of office hours and personal notes of travel, and the like, furnished by the physician himself.)

DR. AND MRS. ALBERT E. PERSONS of West Tupper Street left in July for an extended Western trip, including Yellow Stone Park and Alaska.

---

DR. BENJAMIN H. GROVE of Pearl Street sailed on Thursday, July 13th, on the North American Lloyd steamer George Washington for Bremen. He will tour for a few weeks through Germany.

---

DR. JAMES L. GALLAGHER, Dr. C. J. Carr and Dr. W. H. Billings sailed from Montreal early in July for a tour through Europe, and will return the early part of September.

---

OUR readers will join us in extending sympathy to Dr. Harry A. Wood, whose wife has recently died.

---

DR. FRANCIS E. FRONCZAK has waived, for the remainder of his term of office, the increase of salary from $4,000 to $5,000, passed by the Common Council over the Mayor's veto, in June. We trust this action will be taken as a precedent by officials generally as to the propriety of refusing an increase of salary during an unexpired term of office.

---

DR. GEORGE B. STOCKER spent a week in Cleveland in July, as delegate to the supreme convention of the K. O. T. M.

---

DR. Ernst Fuchs, privy councilor of the University of Vienna, on his way to deliver a course of lectures at Leland Stanford University, was the guest of Dr. Lucien Howe.

---

DR. WILLIAM OSLER of Oxford, received a baronetcy in the distribution of coronation honors. This honor is not merely a personal tribute but a recognition of the value of medical science in general and, indirectly, of medical progress in Canada and the United States. Three years ago, we enjoyed Dr. Osler's hospitality at his home in Oxford. Twenty-three years ago, we gained much from his teaching. Our greetings to Sir William.

---

Dr. C. A. Bentz will spend a year or more in foreign study, at Vienna, Paris, Berlin and Dresden.

---

Dr. John Chalmers attended the convention of the K. O. T. M. in Cleveland in July.

---

Dr. Wm. T. Getman is taking a vacation at Boston.

---

Dr. F. C. Busch is spending two months, investigating problems in comparative physiology and embryology at the U. S. Fisheries Station at East Orland, Me.

---

Buffalo had a small representation at the latest A. M. A. meeting in Los Angeles—Drs. A. A. Jones, F. Parke Lewis, Wm. H. Thornton and Clara A. March.

---

Dr. Wm. P. Clothier is spending a month at Chatauqua.

---

Dr. James A. McLeod reported two cases of extra-uterine pregnancy at the recent annual meeting of the Ontario Medical Association.

---

Dr. Charles Van Bergen is spending a month at Bretton Woods, N. H.

---

Dr. Dewitt H. Sherman visited in the Adirondacks during July.

---

Dr. Wm. F. Gallivan, of the class of 1909, University of Buffalo, who has been house surgeon at Bellevue for the last two years, sailed in July for Germany to continue his studies.

---

Dr. Frank A. Valanti will sail in August for Europe, expecting to study for a couple of months in London and then to pass the winter in Vienna, later attending clinics in some of the Italian cities.

